# A New Role of the Mosquito Complement-like Cascade in Male Fertility in *Anopheles gambiae*

**DOI:** 10.1371/journal.pbio.1002255

**Published:** 2015-09-22

**Authors:** Julien Pompon, Elena A. Levashina

**Affiliations:** 1 CNRS UPR9022, Inserm U963, Université de Strasbourg, Institut de Biologie Moléculaire et Cellulaire, Strasbourg, France; 2 Max Planck Institute for Infection Biology, Berlin, Germany; Stanford University, UNITED STATES

## Abstract

Thioester-containing protein 1 (TEP1) is a key immune factor that determines mosquito resistance to a wide range of pathogens, including malaria parasites. Here we report a new allele-specific function of TEP1 in male fertility. We demonstrate that during spermatogenesis TEP1 binds to and removes damaged cells through the same complement-like cascade that kills malaria parasites in the mosquito midgut. Further, higher fertility rates are mediated by an allele that renders the mosquito susceptible to *Plasmodium*. By elucidating the molecular and genetic mechanisms underlying TEP1 function in spermatogenesis, our study suggests that pleiotropic antagonism between reproduction and immunity may shape resistance of mosquito populations to malaria parasites.

## Abstract 2

The complement-related protein TEP1, which helps kill malaria parasites, also labels damaged cells for removal during mosquito spermatogenesis and promotes male fertility in the malaria vector *Anopheles gambiae*.

## Author Summary

While Anopheline mosquitoes are the most efficient vectors of human malaria, they do have protective mechanisms directed against the causative parasite, *Plasmodium falciparum*. Their immune system targets the invading parasites through activation of the mosquito complement-like system. A central component of this system, thioester-containing protein 1 (TEP1), is a highly polymorphic gene with four allelic classes. Although one class, called R1, mediates efficient parasite elimination, the other classes render the mosquitoes susceptible to *Plasmodium* infections. Until now, it was not clear how or why any of these susceptible *TEP1* alleles were maintained in the population. Here we discover a new role of TEP1 in male fertility. We demonstrate that mosquitoes use the same mechanism—nitration of target surfaces—to flag both damaged sperm and *Plasmodium* cells. Binding of TEP1 to, and removal of, the aberrant sperm is critical to preserve high fertility rates. In the absence of TEP1, accumulation of damaged sperm degrades male fertility. Surprisingly, in spite of the common mechanism of TEP1 activation, distinct alleles of *TEP1* mediate efficient removal of defective sperm and killing of malaria parasites. Our results suggest that pleiotropic function in immunity and reproduction is one of the mechanisms that maintain *TEP1* polymorphism in mosquito populations.

## Introduction

*Anopheles gambiae* mosquitoes are the most efficient vectors of human malaria. Mosquitoes actively respond to *Plasmodium* infections by mounting immune responses that destroy the majority of invading parasites. These responses are largely mediated by thioester-containing protein 1 (TEP1) [1–4], a homologue of the mammalian complement factor C3. TEP1 is synthesized in the mosquito blood cells and is secreted into the blood or hemolymph, where a protein cascade called “mosquito complement-like system” tightly controls its activity. A series of studies on TEP1-mediated killing of *Plasmodium* parasites demonstrated that a complex of two leucine-rich repeat proteins, leucine-rich immune protein 1 (LRIM1) and *Anopheles Plasmodium*-responsive leucine-rich protein 1C (APL1C), prevents precocious activation of TEP1 [5,6], whereas heme peroxidase 2 (HPX2) and NADPH oxidase 5 (NOX5) oxidases direct TEP1 binding to *Plasmodium* by modifying ookinete surfaces [7]. Elimination of any of these factors does not affect *TEP1* expression but abolishes its binding to parasites and increases mosquito susceptibility to infections [2,7–9]. However, TEP1 function was only examined in the immune responses of females, which are responsible for malaria transmission.

Here we report a new function of TEP1 in male fertility. We demonstrate that TEP1 and other members of the complement-like cascade are present in the testes and uncover an allele-specific *TEP1* contribution to clearance of apoptotic cells during spermatogenesis. We also show that TEP1 binding to defective sperm cells is regulated by the same complement-like cascade that kills malaria parasites in the mosquito midgut. In spite of these similarities, our results demonstrate that male fertility is promoted by the TEP1*S2 allele, which renders mosquitoes susceptible to *Plasmodium* infections. By elucidating the molecular and genetic mechanisms underlying TEP1 function in reproduction, our study reveals an example of pleiotropic antagonism between alleles that may impact the genetic makeup of the mosquito resistance to *Plasmodium*.

## Results

### TEP1 Occurrence in the Mosquito Testis

**Fig 1:**
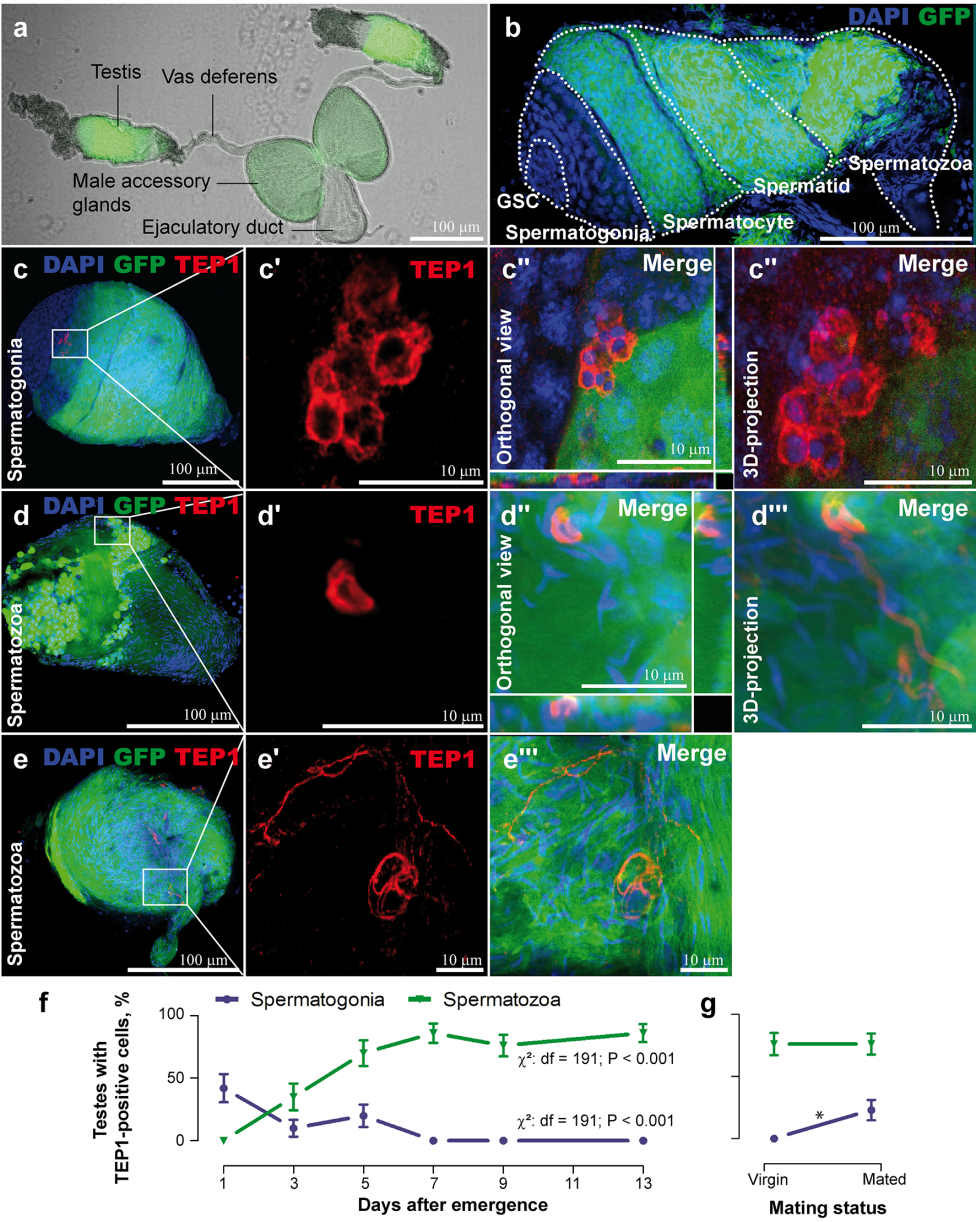
TEP1 occurrence in the testes during spermatogonial development. The *DSX* transgenic line [12] that expresses *GFP* (green) under the *β-tubulin* promoter in the meiotic stages starting from spermatocytes but not in the mitotic germline stem cells (GSC) and spermatogonia. Nuclei were colored with DAPI (blue). (A) Male reproductive organs. (B) Organization of spermatogonial compartments in the testis (dotted lines). (C–E) TEP1 (red) recruitment to the spermatogonia (C–C”‘) and to the spermatozoa’s head (D–D”‘) and tail (E–E”). (F) Occurrence of testes with TEP1-positive cells during the first week after adult emergence. Testes were dissected for immunofluorescence analyses at the indicated time points. (G) Effect of mating on the percentage of testes with TEP1-positive cells. Virgin males (7-d-old) were collected in copula, and 2 d later their testes were dissected for immunofluorescence analyses. Significant differences (*p* < 0.05, χ^2^ test) are shown by an asterisk. Vertical bars show standard deviation, *n* ≥ 30 testes. Data used to make this figure can be found in S1 Data.

Using polyclonal antibodies and confocal microscopy of dissected whole mount testes, we observed TEP1 signal in the spermatogenic compartments of *A*. *gambiae*. The mosquito spermatogenic compartments are distinguished by the shape of the sperm nuclei [10] and by the expression pattern of *β-tubulin* [11]. The apical side of the testes contains a ring of hub cells, the niche of the germline stem cells (GSC) and somatic stem cells (SSC). Upon division, GSCs differentiate into the primary spermatogonia. These compartments do not express the *β-tubulin* gene whose expression begins in the spermatocytes. All sperm cells from the hub to the spermatocytes display rounded nuclei, whereas the nuclei of the spermatids and spermatozoa adopt their mature elongated shape. To facilitate mapping of TEP1-positive cells in the testes, we made use of the *DSX* transgenic line in which *β-tubulin* gene promoter directed the expression of green fluorescent protein (GFP) reporter in spermatocytes, spermatids, and spermatozoa (Fig 1A and 1B) [12]. TEP1 signal was detected on spermatogonia (round nuclei, no expression of *β-tubulin*::*eGFP*, Fig 1C) and on spermatozoa (elongated nuclei, expression of *β-tubulin*::*eGFP*, Fig 1D and 1E). Given this surprising localization, we tested whether TEP1 expression correlates with spermatogonial development, which in mosquitoes is initiated at the larval stages and is completed a couple of days after the emergence of adults [10,13]. In *Anopheles* males, copulation triggers a new wave of spermatogenesis to replenish the ejaculated spermatozoa [10]. Therefore, we monitored the occurrence of TEP1 signal in the testes of virgin males during the first 2 wk after adult emergence and after mating. The percentage of testes with TEP1-positive spermatogonia decreased during the first week after male emergence and correlated with the termination of spermatogenesis (Fig 1F). Consistent with the onset of sperm production, the proportion of testes with TEP1-positive spermatogonia increased after mating (Fig 1G). In contrast, spermatogenesis did not promote the occurrence of TEP1-positive spermatozoa, whose numbers were increasing with time after adult emergence. These data suggest that TEP1 occurrence in the testes correlates with spermatogenic development.

### The Conserved Complement-like Cascade Directs TEP1 Binding to Damaged Sperm for Removal

**Fig 2:**
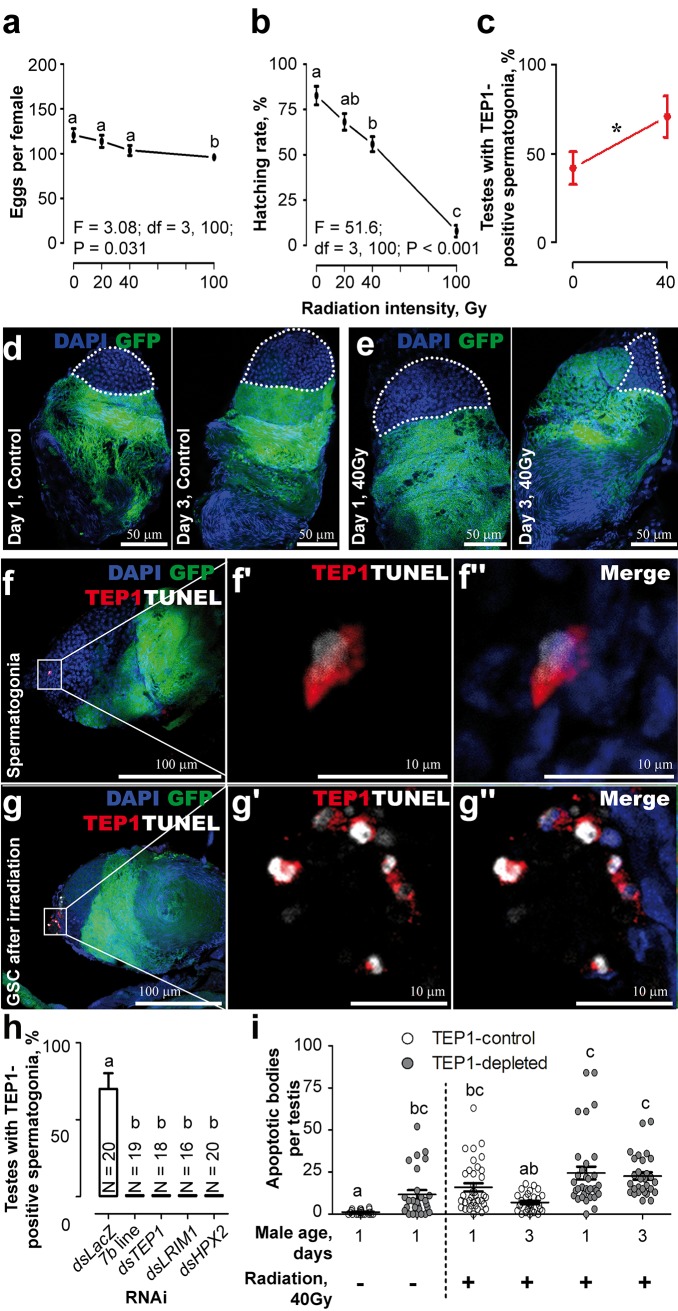
Effect of radiation on TEP1-mediated removal of damaged sperm. A *DSX* transgenic line expressing *GFP* (green) under the *β-tubulin* promoter was used. Nuclei were colored with DAPI (blue). (A,B) 3-d-old virgin males that emerged from irradiated pupae were mated with 3-d-old virgin females, and the number of (A) laid eggs and the (B) larval hatching rates per female were gauged. Means ± standard error of the mean (SEM) are plotted for *n* ≥ 25. (C) The proportion of testes with TEP1-positive spermatogonia in irradiated males (40 Gy), *n* ≥ 30. (D,E) Radiation (40 Gy) reduces the size of the spermatogonial compartment (white dotted line) in 1- and 3-d-old males. (F,G) Colocalization of TEP1 (red) and TUNEL (white) signals in (F–F”) spermatogonia and (G–G”) the GSC in irradiated 1-d-old males. (H) Occurrence of TEP1 in spermatogonia of irradiated (40 Gy) males (*DSX)* injected with *dsTEP1*, *dsLRIM1*, *dsHPX2*, and *dsLacZ* (control). Males depleted for *TEP1* (*7b* line) served as positive controls. The proportion of testes with TEP1 signal was gauged 2 d later. Mean ± standard error (SE) is shown; N, number of testes. (i) Accumulation of TUNEL-positive spermatogonia after irradiation in the testes of control and TEP1-depleted males (progeny of reciprocal crosses between *7b* and *DSX*) was examined 1 and 3 d after emergence. Each dot represents one testis. Significant differences (*p* < 0.05, χ^2^ test) are shown by an asterisk and by characters above the corresponding values. Data used to make this figure can be found in S1 Data.

As spermatogenesis is accompanied by waves of apoptosis [14,15], we asked whether TEP1 was recruited to defective spermatogonia. To this end, massive sperm damage was experimentally induced in *DSX* pupae by radiation [10,16]. We first gauged the impact of radiation on male fertility and confirmed that it drastically decreased hatching rates of the progeny (Fig 2A and 2B). A significant reduction in egg laying was only observed in females mated with the *DSX* males irradiated with the highest dose (100 Gray [Gy]). Further, we observed a significant increase in the proportion of testes with TEP1-positive spermatogonia, which correlated with the reduction in the size of the spermatogonial compartment in 3-d-old irradiated *DSX* males (Fig 2C–2E, S1 Fig), suggestive of TEP1 recruitment to defective cells. Indeed, radiation significantly increased the number of damaged cells, as measured by TdT-mediated dUTP nick-end labeling (TUNEL), which marks DNA breaks associated with cell death. Interestingly, all TEP1-positive cells in the testes of control and irradiated males were TUNEL positive, but not all TUNEL-positive cells were also stained with the anti-TEP1 antibody (Fig 2F). Moreover, radiation-induced cell damage and TEP1 signal were also detected in the GFP-negative germ-line compartment (Fig 2G). These results suggest that TEP1 is recruited to damaged cells.

The specificity of the detected TEP1 signal was confirmed by two independent approaches. First, we used a mosquito transgenic line (*7b*) in which *TEP1* expression is constitutively repressed by a dominant transgene-mediated silencing (S2 Fig). Second, we injected irradiated *DSX* males with double-stranded RNA (dsRNA) against *TEP1* 1 d after emergence. TEP1 signal was not detected in the testes of irradiated males in both TEP1 depletion methods, confirming signal specificity (Fig 2H, S2 Fig). We next examined whether other members of the complement-like cascade were present in the testes, using polyclonal antibodies against LRIM1 and HPX2 and confocal microscopy. Neither LRIM1 nor HPX2 signals associated with the sperm cells, but they were detected in the cells attached to the testes (S3 Fig). Since elimination of any of these factors does not affect TEP1 expression but abolishes TEP1 binding to parasites [2,7–9], we exploited their silencing to discriminate whether TEP1 is expressed in or bound to the sperm cells. Depletion of either protein abolished TEP1 signal on spermatogonia but did not affect TEP1 expression, as evidenced by a clear TEP1 signal detected by immunoblotting in the hemolymph extracts of males (Fig 2H, S4 Fig), supporting the hypothesis that the observed signal resulted from TEP1 binding to spermatogonia. Taken together, these results demonstrate that the complement-like cascade regulates TEP1 binding to defective sperm.

In mammals, complement contributes to the removal of apoptotic cells [17]. Therefore, we examined whether TEP1 binding mediated clearance of defective spermatogonia by comparing the number of TUNEL-positive cells in the testes of control and TEP1-depleted males in a similar genetic background of the F_1_ progeny of a reciprocal cross between *DSX* and *7b* (S5 Fig). Significantly higher numbers of TUNEL-positive cells were detected in the absence of TEP1 in the testes of freshly emerged males (Fig 2I, day 1). Furthermore, while irradiation increased the number of damaged cells in all samples, their numbers decreased with time in controls but remained high in TEP1-depleted mosquitoes (Fig 2I, day 3). These results suggest that TEP1 binding to the spermatogonia correlates with removal of defective cells during spermatogenesis.

### TEP1 Rescues Radiation-Induced Male Infertility

**Fig 3:**
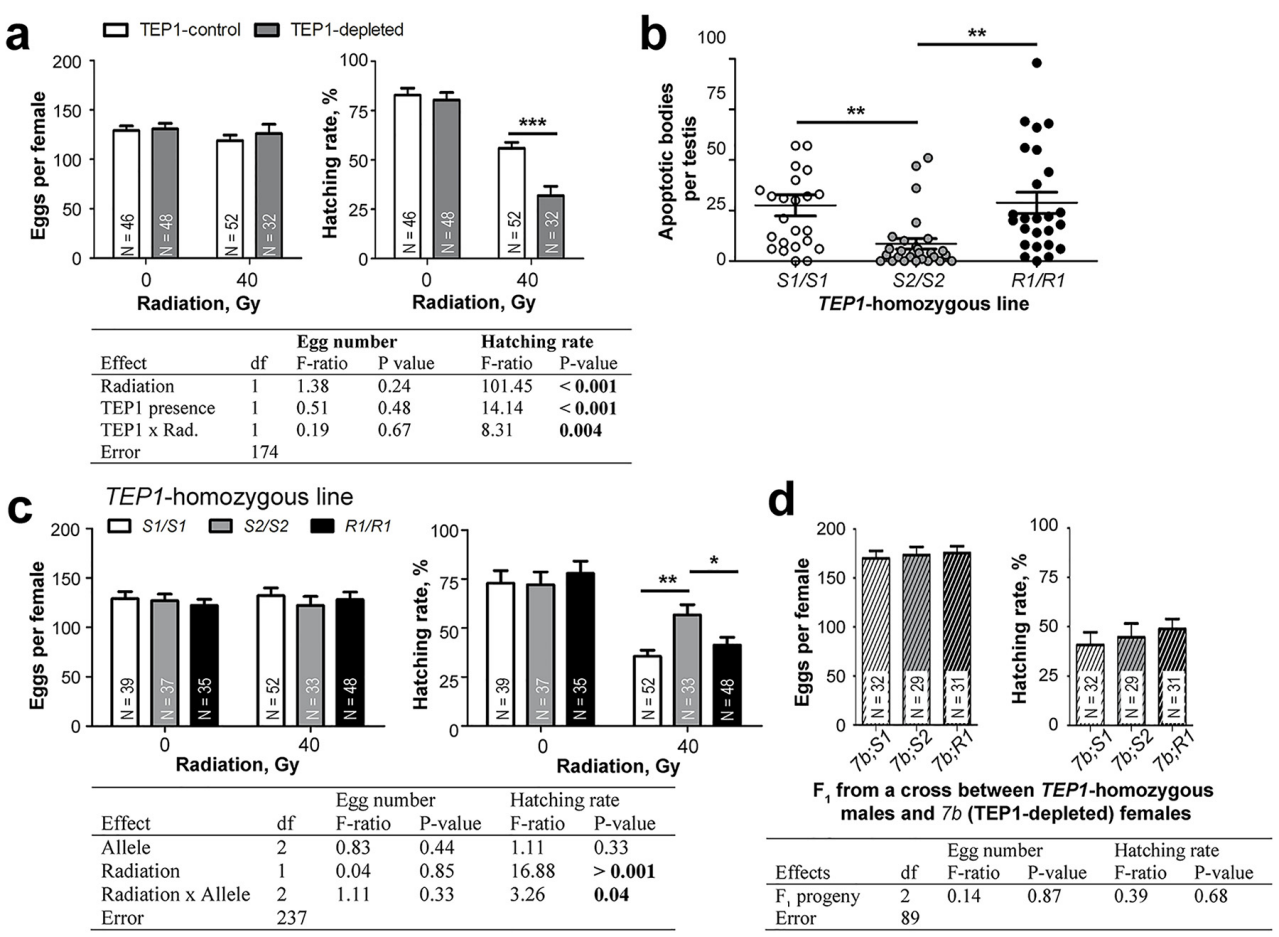
Effect of radiation and TEP1 depletion on male fertility. Pupae were irradiated (40 Gy), and the resulting 3-d-old males were mated with 3-d-old females. The mean ± SEM of laid eggs and the proportions of hatched larvae are plotted. N, number of oviposited females. (A) After irradiation, TEP1 depletion (*7b* line) decreases hatching rates as compared to controls (*T4* line). (B) The proportion of testes with apoptotic cells was examined by TUNEL staining in irradiated *TEP1-*homozygous (*S1*/*S1*, *S2*/*S2*, or *R1*/*R1*) 1-d-old males. Each dot represents one testis. (C) Irradiated *TEP1*-homozygous (*S1*/*S1*, *S2*/*S2*, or *R1*/*R1*) 3-d-old males were mated with TEP1*S1/S1 females. (D) *TEP1* expression was silenced in the males of F_1_ reciprocal crosses between *7b* and each of the *TEP1*-homozygogus lines. Irradiated F_1_ 3-d-old males were mated with TEP1*S1/S1-homozygogus females. The results of two-way analysis of variance (ANOVA) tests are shown in tables below the corresponding graphs. Post hoc Tukey’s test: * *p* < 0.05; ** *p* < 0.01; *** *p* < 0.001. Data used to make this figure can be found in S1 Data.

Based on these results, we proposed that TEP1 marks damaged spermatogonia for removal. Accumulation of dead cells degrades sperm quality [18]. Therefore, we hypothesized that TEP1 deficiency should decrease male fertility after irradiation. Indeed, radiation significantly decreased larval hatching rates (Fig 3A). Importantly, a larger decrease was observed in the progeny of TEP1-depleted males as compared to controls, suggesting that TEP1 partially rescues radiation-induced male sterility.

*TEP1* is a highly polymorphic gene with four major alleles that differ in their capacity to kill or to resist malaria parasites—namely, the resistance-associated alleles *R1* and *R2* and the susceptibility-associated alleles *S1* and *S2* [19–22]. Resistance alleles are believed to offer selective advantages to the mosquitoes infected with *Plasmodium*; however, the fitness costs of these alleles have not been experimentally tested. To explore the impact of *TEP1* polymorphism on the fertility of irradiated males, we generated three *S1*/*S1*, *S2*/*S2*, and *R1*/*R1* homozygous lines from the *TEP1*-heterozygous *T4* line of *A*. *gambiae* obtained from Imperial College, London (S6 Fig). Indeed, *TEP1* genotyping of this line identified the following frequencies of *TEP1* alleles: *R1* (14%), *S1* (67%), and *S2* (19%). To obtain *TEP1* homozygous lines, the legs of virgin females and males were used for nested PCR-restriction fragment length polymorphism (RFLP) genotyping, and the reciprocal crosses were set up between the selected individuals. Once the *TEP1* homozygosity of the established lines was confirmed, male pupae from each line were irradiated, and the testes of the resulting males were dissected, stained, and microscopically examined. Lower numbers of TUNEL-positive cells were observed in the testes of *S2*/*S2* as compared to *S1*/*S1* and *R1*/*R1* males (Fig 3B). Moreover, the progeny of *S2*/*S2* males had higher hatching rates than *S1*/*S1* and *R1*/*R1* irradiated males (Fig 3C). Importantly, no differences were observed between these lines in the absence of radiation. These results suggest that the TEP1*S2 allele efficiently protects against radiation-induced male sterility. To exclude the possibility that the observed differences were caused by variation at an unrelated locus, we generated heterozygous *S1*, *S2*, and *R1* males depleted for TEP1 by crossing *TEP1* homozygous males from each of the three lines with *7b* females (S6 Fig). Pupae of the resulting progeny were irradiated, and the obtained TEP1-depleted 3-d-old males were crossed with TEP1*S1-homozygous females. Similar sizes of egg batches and egg-hatching rates indicated that TEP1 depletion abrogated fertility advantages of the *S2* allele (Fig 3D). Taken together, our results indicate the allele-specific contribution of *TEP1* to higher male fertility rates in stressful conditions induced by radiation.

### Allele-Specific Role of *TEP1* in Male Fertility

**Fig 4:**
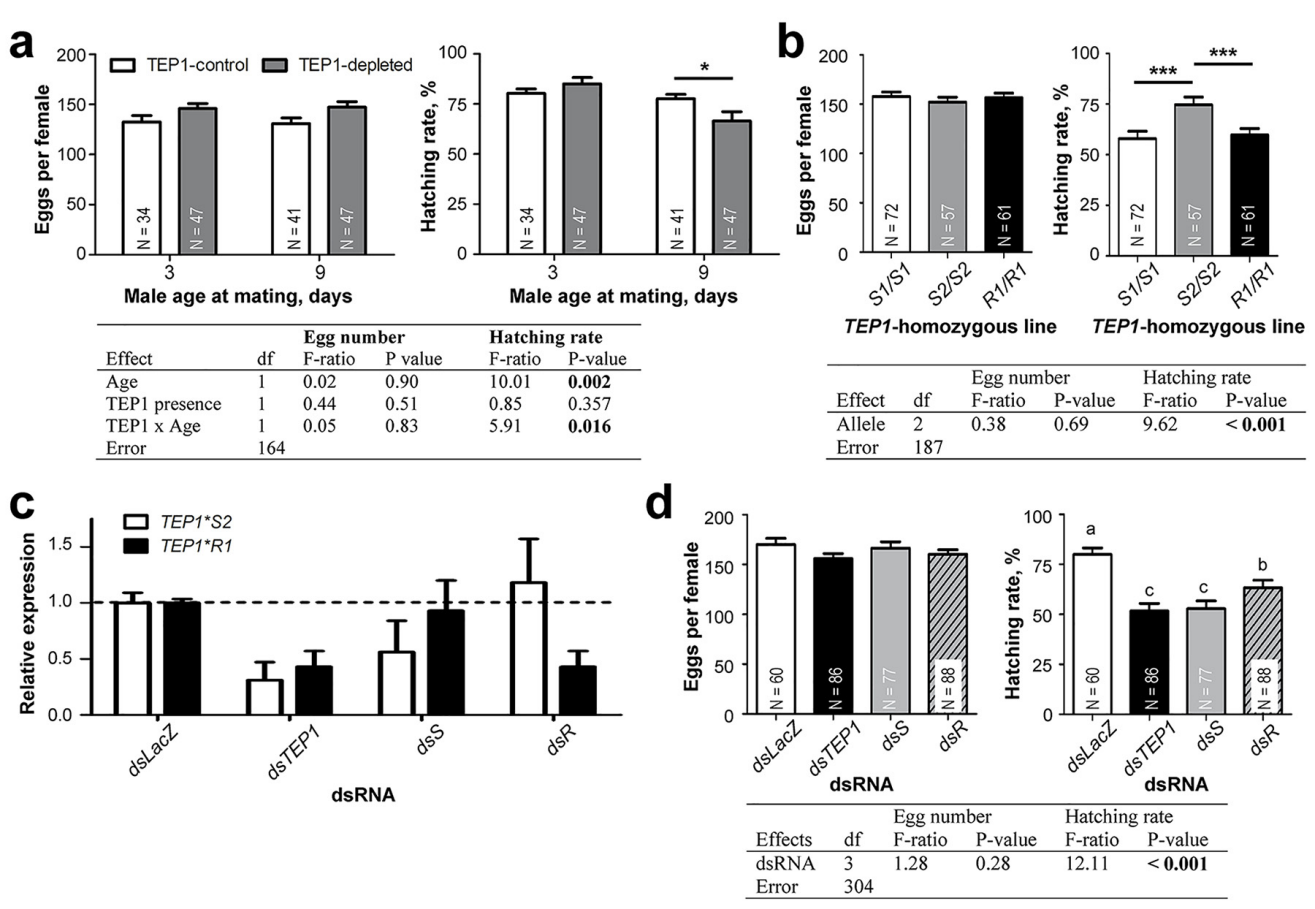
Allele-specific function of TEP1 in male fertility. Males (3- and 9-d-old) were mated with 3-d-old females. The mean ± SEM of laid eggs and the proportions of hatched larvae are plotted. N, number of oviposited females. (A) Fertility rates of TEP1-depleted (*7b* line) and TEP1-control (*T4* line) males. (B) The fertility of *TEP1*-homozygous (*S1*/*S1*, *S2*/*S2*, or *R1*/*R1*) males mated with TEP1*S1/S1 females. (C) 1-d-old TEP1*S2/R1 males were injected with dsTEP1*S2- (*dsS*) or with dsTEP1*R1-(*dsR*), and 12 d later, relative expression levels of *TEP1* were gauged by allele-specific quantitative reverse transcription PCR (qRT-PCR). Injection of *dsLacZ* and *dsTEP1* served as a negative and a positive control, respectively. Expression of a gene encoding ribosomal protein L19 (*RpL19*) was used for normalization. Results of three independent experiments are plotted. (D) 1-d-old TEP1*S2/*R1* males were injected with dsTEP1*S2- (*dsS*) or with dsTEP1*R1-(*dsR*) and mated 8 d later with TEP1*S1/*S1* females. Injection of *dsLacZ* and *dsTEP1* served as a negative and a positive control, respectively. Results of two-way ANOVA tests are shown in tables below the corresponding graphs. Statistically significant differences in (A,B): * *p* < 0.05; *** *p* < 0.001, post hoc Tukey’s test, and in (D): *p* < 0.05 (Fisher’s LSD test), are indicated by characters above the corresponding values. Data used to make this figure can be found in S1 Data.

We next evaluated whether TEP1 regulated male fertility under normal conditions. The radiation experiments described above were performed with young males (3-d-old). However, age plays a critical role in male mating behavior and insemination success. Although 3-d-old males are sexually competent, mosquito males show the highest levels of mating activity and form mating swarms several days later (7 to 9 d after emergence) [23,24]. Therefore, we compared the fertility of young (3-d-old) and mature (9-d-old) control and TEP1-depleted males. Moderate but significantly lower hatching rates were observed in the progeny of mature but not young TEP1-depleted males as compared to same-age controls (Fig 4A), demonstrating that TEP1 regulates male fertility in the absence of radiation. To make sure that the observed phenotype was due to clearance of damaged sperm cells and not a consequence of microbial infections in TEP1-depleted mosquitoes, we examined bacterial loads in the testes and male accessory glands (MAGs) of control and TEP1-depleted 1- and 13-d-old mosquitoes by quantitative PCR of the conserved bacterial *16S* rRNA gene. No significant differences between the two groups were detected (S7 Fig), indicating that TEP1 depletion did not cause significant changes in microbiota proliferation in male reproductive tissues that could explain the observed impact of TEP1 depletion on male sterility.

To validate the specific contribution of the TEP1*S2 allele to male fertility, we compared the fertility rates of mature males of the *TEP1 S1/S1*, *S2/S2*, or *R1/R1* homozygous lines described above. As in the radiation experiments, significantly higher hatching rates were detected in the progeny of *S2*/*S2* males (Fig 4B). The causative effect of the *TEP1* polymorphism was further validated by reciprocal allele-specific RNA interference, which interrogates the effects of allele-specific silencing in an identical genetic background [21]. To this end, we performed reciprocal crosses between *R1*/*R1* and *S2*/*S2* homozygous lines and used males of F_1_ progeny heterozygous for *TEP1* (*S2*/*R1)* for injections with allele-specific dsRNAs targeting *S2* (*dsS*) or *R1* (*dsR*). Injections of *dsLacZ* and *dsTEP1* served as negative and positive controls, respectively. A significant reduction in the expression of targeted alleles was confirmed by quantitative PCR (Fig 4C). A moderate but significant decrease in hatching rates was detected in the progeny of *dsS-* and *dsTEP1*-depleted males (Fig 4D), thereby confirming the role of the *S2* allele in male fertility. We noted that the progeny of *dsR-*injected males displayed reduced hatching rates as compared to control *dsLacZ*, pointing towards epistatic interactions between *TEP1* alleles. Taken together, these data demonstrate the contribution of TEP1*S2 allele to male fertility, thereby raising a possibility of a fitness trade-off between *TEP1* alleles in reproduction and immunity.

## Discussion

We discovered a new function of the complement-like cascade in spermatogenesis that impacts male fertility in the malaria mosquito. We demonstrate that depletion of the complement-like factor TEP1 results in accumulation of defective sperm and decreases male fertility. Our study extends the role of the mosquito complement-like cascade from immunity to the removal of defective sperm during spermatogenesis and, thereby, to male fertility and reproduction.

We also show that TEP1 function in spermatogenesis is regulated by the same HPX2-LRIM1 axis that controls killing of *Plasmodium* parasites in the midgut. We propose that activation of the TEP1/LRIM1/APL1C complex is induced by an unknown mechanism in the proximity of the damaged sperm cells. It appears that the surface modification mediated by HPX2 is required for TEP1 binding to the damaged sperm, suggesting that the same signals are used by the mosquitoes to label defective cells and invading pathogens (S8 Fig). This conserved requirement for reactive nitrogen species in the activation of the mosquito complement-like system in immunity and reproduction suggests that nitration through oxidation of cell surface proteins may be a general mechanism of complement activation, which may be relevant for the activation of the alternative complement pathway in mammals (S8 Fig). The role of the complement factor C3, a mammalian homologue of TEP1, in the clearance of apoptotic bodies is well documented [17,25]. However, C3 deficient mice are fully fertile, and sperm quality is kept in check by a complement-independent mechanism, probably to prevent a deleterious complement activation on self surfaces and gamete damage [26]. Future studies should examine the mechanism(s) that mediate(s) removal of damaged cells and of invading parasites. In *Drosophila*, electron microscopy detected traces of the abnormal sperm in the neighboring cells but also inside the macrophage-like cells present in the testis tissues [27]. Although phagocytosis may contribute to the removal of the damaged sperm cells, this process is irrelevant for parasite clearance in the midgut, as no phagocytic cells have been detected by electron microscopy [28].

Previous reports demonstrated the causative role of the *R1* allele in mosquito resistance to infections with *Plasmodium berghei* and with some isolates of *P*. *falciparum* [21,22,29,30]. However, low rates of infected mosquitoes in natural populations question the impact of *Plasmodium* on *TEP1* evolution. Given the broad function of TEP1 in immune responses to bacteria and fungi [19,31–33], local microbial pressures on the larval stages may drive its exceptional variability across Africa [22], a hypothesis that has never been experimentally proven. Here we show that *TEP1* affects reproduction at the age when males actively engage in mating, suggesting that our findings are relevant for male fitness in natural mosquito populations. Moreover, we report that in contrast to resistance to *Plasmodium*, a distinct “susceptible” *S2* allele mediates higher male fertility rates. At the structural level, the S2 form of TEP1 is mostly similar to the S1 form, except for a β-hairpin loop, shared with R1 and R2 and located on the convex surface of the thioester domain [34]. The exposed position of this loop is suggestive of its role in protein–protein interactions. Although interactions with distinct partners may define a critical role of S2 form in cell removal, further studies are needed to examine biochemical bases of *TEP1* pleiotropism. A recent report demonstrated the role of the mosquito heme peroxidase 15 (HPX15) in long-term female fertility by protecting sperm from oxidative damage during storage in spermathecae [35]. The results presented here uncover the role of the HPX2/TEP1/LRIM1 complement-like cascade in the removal of defective sperm cells in the testes, the process that promotes male fertility rates. As strong selective pressures apply to genes involved in reproduction [36] and male fitness [37], even the moderate contribution of *S2* to male fertility revealed in our experiments may have important consequences for mosquito field populations, as enrichment in *S2* alleles may render mosquito populations more susceptible to *Plasmodium* infections. Taken together, we propose that pleiotropic antagonism may drive the evolution of the *TEP1* locus and shape the genetic makeup of resistance to *Plasmodium* in a major malaria vector.

## Methods

### Ethics Statement

All work in this study was performed in agreement with national animal work authorization E 67-484-2 issued by the Department of Veterinary Services, Prefecture du Bas-Rhin, France.

### Mosquito Strains

*A*. *gambiae* sensu stricto strain G3 and *TEP1* homozygous and transgenic lines originating from it were used throughout the study. Mosquitoes were reared in the insectary at 28 ± 2°C and 80 ± 5% humidity with a 12/12 h dark/light cycle. Larvae were fed with grinded fish food (Tetra), and adults received a 10% sugar solution (w/v). All work in this study was performed in agreement with national animal work authorization E 67-482-2 issued by the Department of Veterinary Services, Prefecture du Bas-Rhin, France.

#### Transgenic lines

The *DSX* line (TEP1*S1/S1) expressed three reporters: (i) a *3xP3*::*DsRed* transgenesis marker in the mosquito nervous system [38]; (ii) a *β-tubulin*::*eGFP* reporter expressed in spermatocytes, spermatids, and spermatozoa; and (iii) a *DmActin5C*::*eGFP* reporter expressed predominantly in the midgut [12]. *DSX* mosquitoes were used to differentiate spermatogenic compartments in the testes.

The *7b* line (TEP1*S1/S1) was obtained by a piggyBac-mediated transgene insertion on the X chromosome. The transgene contains (i) a *3xP3*::*DsRed* transgenesis marker expressed in the mosquito nervous system [38] and (ii) a *DmHsp70*::*TEP1* cassette that causes a dominant depletion of TEP1 (S2 Fig). *7b* mosquitoes were used as a positive control for experiments testing the effect of TEP1 depletion on spermatogenesis and on male fertility, while the transgenesis *3xP3*::*DsRed* marker of this line inserted on the X chromosome was used to sex larvae at an early stage, using a COPAS instrument (Union Biometrica) as described [39] to ensure adults’ virginity.

The *T4* line, obtained from the Imperial College, London, expressed the *3xP3*::*eGFP* reporter in the nervous system of males but not females because of the piggyBac transposon insertion on the Y chromosome [40]. This line was used for high-throughput male selection using the COPAS instrument. Genotyping of the *T4* line showed that it was heterozygous for *TEP1* and contained *R1* (14%), *S1* (67%), and *S2* (19%) alleles; therefore, we used this line for establishment of the *TEP1* homozygous lines.

#### *TEP1* homozygous lines

To establish *TEP1*-homozygous lines from the heterozygous *T4* population, legs from 192 live males and 192 live females were genotyped by the PCR-RFLP method described below. Individuals (25 males and 25 females) with the selected genotypes for each homozygous line were used for crosses. After three rounds of intracrossing, the resulting TEP1*S1/S1, *S2/S2*, and *R1/R1* lines were established and used for experiments within the next five generations.

### Genetic Crosses

F_1_ progenies of the reciprocal crosses between *DSX* and *7b* lines were used for experiments with *7b*; *DSX* and wt; *DSX* males. To obtain *7b*/*S1*, *7b*/*S2*, and *7b*/*R1* heterozygous males, F_1_ progenies of crosses between *7b* females and TEP1*S1/*S1*, *S2/*S2*, and *R1/*R1* homozygous males were used. Males heterozygous for TEP1*S2/*R1* were from the F_1_ progeny of a cross between TEP1*R1/R1 females and TEP1*S2/S2 males. The COPAS instrument (Union Biometrica) was used for sexing of the early larval stages [39]. Females and males of *DSX*, *TEP1*-homozygous, and *7b* lines were raised separately, and 100 males and 50 females were mated in each cross.

### *TEP1* Genotyping

To identify *TEP1* alleles in the mosquito lines used in this study, we used a nested PCR-RFLP. DNA was extracted from one mosquito leg in 40 μl of grinding buffer (10 mM Tris-HCl pH 8.2; 1 mM EDTA; 25 mM NaCl) mixed with 0.4 μl of 100x Proteinase K (Sigma) and incubated for 45 min at 37°C. A first PCR was conducted using VB3 5ʹ-*ATGTGGTGAGCAGAATATGG*-3ʹ and VB4 5ʹ-*ACATCAATTTGCTCCGAGTT*-3ʹ primers, followed by a second PCR performed on 2 μl of the resulting product with AG1656 5ʹ-*ATCTAATCGACAAAGCTACGAATTT*-3ʹ and AG1653 5ʹ-*CTTCAGTTGAACGGTGTAGTCGTT*-3ʹ primers, producing a final fragment of 764 bp. Both PCR reactions were subjected to 95°C for 2 min, 30 cycles with 15 s at 95°C, 15 s at 55°C, and 45 s at 72°C, and a final step at 72°C for 5 min, using GO Tag Green Master mix (Promega). PCR products were digested by *Bam* HI, *Hind* III, or *Bse* NI (Fermentas) and analyzed on 1.5% agarose gels. Expected restriction patterns are provided in S1 Table. Genotyping was conducted on 96 males and 96 females from *T4*, *DSX*, *7b*, and on each of the *TEP1*-homozygous and *TEP1*-heterozygous lines.

### *TEP1* Sequencing

Verification of *TEP1* sequences from homozygous lines was obtained by amplicon sequencing as described [21] using DNA extracts of five different mosquitoes for each line. DNA was extracted using the DNeasy kit (QIAGEN). Sequencing revealed that TEP1*S1 corresponded to the TEP1*S4 allele initially identified in the *VKper* line, TEP1*S2 to the TEP1*S6 allele from the Ngousso, and TEP1*R1 to the TEP1*R1 from the L3-5 refractory line [21,29].

### Immunofluorescence and Confocal Microscopy

To examine the presence of TEP1, HPX2, and LRIM1, the testes were dissected in 1x PBS, fixed with 2% PFA in 1x PBS for 1 h; permeabilized with 0.5% Triton in 1x PBS for 20 min; blocked with 0.5% Triton, 1% BSA, in 1x PBS for 30 min; incubated overnight at 4°C with anti-TEP1 (1/300) [41], the anti-HPX2 [7], or anti-LRIM1 [5] polyclonal rabbit antibodies in the blocking solution and then washed with 0.5% Triton, 1% BSA, in 1x PBS; and incubated for 2 h with a secondary fluorescence-labeled Cy3 or Cy5 anti-rabbit IgG antibody (1/1,000) (Jackson Laboratory) and DAPI (1/5,000) (Vector Laboratories) in the blocking solution for 15 min.

To reveal DNA damage, the TUNEL assay was performed on TEP1-stained testes dehydrated for 3 h on SuperFrost/Plus slides (Menzel-Glazer) using the ApopTag Red *In Situ* kit (Millipore).

To determine TEP1 occurrence in the testes after mating, 2-d-old virgin males were added to a cage containing virgin females, and couples were collected *in copula* within the first hour. Males were then kept in a separate cage for 2 d. The testes were dissected and assessed for TEP1 presence using anti-TEP1 polyclonal antibodies and immunofluorescence.

All samples were mounted in Vectashield medium (Vector Laboratories), examined using a LSM700 laser confocal microscope (Zeiss), and analyzed using Image J open source software [42] with the Figure J package [43].

### Immunoblotting

To determine the presence of TEP1, LRIM1, or HPX2 in the hemolymph or the testes, hemolymph samples from ten mosquitoes were collected by proboscis clipping, and the testes extracts were obtained by grinding 20 dissected testes in 10 μl of Laemmli buffer (Tris-HCl 0.35 M; 10.3% SDS; 36% glycerol; 5% β-mercaptoethanol; 0.012% bromophenol blue). Extracts were separated by precast 4%–20% gradient SDS-PAGE (Bio-Rad). Protein membrane transfer, antibody incubations, and detection were carried out, as previously described [41], using anti-TEP1 [41] (1/1,000) and anti-LRIM1 [5] antibodies (1/300), with anti-prophenoloxidase 2 (PPO2) (1/15,000) or anti-α-actin antibody (1/1,000) (Chemicon) as loading controls. Immunoblotting of HPX2 was conducted in native conditions as described [7]. Bound antibodies were detected by an anti-rabbit or anti-mouse IgG conjugated to horseradish peroxidase (1/30,000) (Promega) using Super Signal West Pico Chemiluminescent Substrate (Thermo Scientific).

### RNAi Silencing

To deplete TEP1, LRIM1, TEP1*S, or TEP1*R, dsRNAs for *TEP1*, *LRIM1*, *LacZ*, TEP1*S (*dsS*), and TEP1*R (*dsR*) were produced from plasmids containing the dsRNA-target sequence flanked by two *T7* promoters [5,21,44]. To deplete HPX2, dsRNA for *HPX2* was produced from PCR-amplified fragments flanked with two *T7* promoters as described [7]. RNA synthesis and purification were performed using MegaScript and MegaClear kits (Ambion). RNA concentrations were measured by Nanodrop (Thermo Scientific) before annealing 3 μg/μl of sense and anti-sense RNAs by boiling. DsRNAs were injected (69 nl) into the thorax of CO_2_-immobilized 1-d-old mosquitoes using a glass capillary mounted onto a Nanoject II injector (Drummond).

### Quantitative Reverse Transcription PCR

To quantify the mRNA level for *TEP1*, *LRIM1*, and *HPX2*, total RNA was extracted from ten mosquitoes using the RNeasy extraction kit (QIAGEN) or RNAzol (Sigma-Aldrich) and reverse-transcribed by the M-MuLV Reverse Transcription kit (Thermo Scientific). Gene expression was quantified using primers and probes detailed in S2 Table by quantitative PCR with Fast SybrGreen chemistry for *LRIM1* and *HPX2* and TaqMan chemistry for *TEP1* and *RPL19*, using the ABI 7500 Fast Real-Time PCR machine. Expression of *RPL19*, the gene encoding housekeeping ribosomal protein L19, was used for normalization.

### γ-Ray Irradiation

To induce sperm damage, pupae were subjected to radiation within the first 12 h after pupation using a Biobeam 8000 (Gamma-Service Medical Research).

### Fertility Assays

To estimate the impact of TEP1 presence or the *TEP1* allele on male fertility, female and male larvae were sexed by the COPAS instrument and raised separately [39]. On day 3 after emergence, 25 virgin females were mixed with 50 virgin males in a cubic 17 cm cage. Females were fed on a mouse 5 d later, and unfed females were removed from the cage. On day 7, individual females were placed into plastic vials containing 1–2 ml of water and closed with cotton pads. On days 9–10, the deposited eggs and larvae were counted in each vial. We confirmed that females that did not lay eggs had no sperm in their spermathecae, as previously reported [45]. For fertility assays with mature males, males were kept virgin, and on day 9 after emergence, they were mated to 3-d-old females and assessed as above. Experiments were repeated four times: two independent experiments with two different cages.

### Estimation of Bacterial Loads

To examine the effect of TEP1 depletion on bacterial loads in the male sexual organs, control (*T4)* and TEP1-depleted (*7b*) larvae were reared in the same water to ensure equal exposure to the environmental microbes. The adults were separated after eclosion based on expression of fluorescent markers. On days 1 and 13 after emergence, the testes and MAGs were dissected from five adults per group, and DNA was extracted using the DNeasy kit (QIAGEN). Bacterial loads were gauged by quantitative PCR of the highly conserved *16S* rRNA gene (S2 Table) [46]. Three independent biological experiments were conducted.

### Statistical Analysis

Normalization of the distribution and the homogeneity of variances was evaluated by Shapiro’s and Bartlett’s tests, respectively. To fit normal distribution and homogeneity of variances, larval hatching rates were arcsin square root-transformed before analysis. Statistical analyses were conducted using SYSTAT 12.0 (SYSTAT software) and R (http://www.R-project.org) software.

## Supporting Information

S1 Data Raw data used to generate plots in Figs 1F, 1G, 2A–2C, 2H, 2I, 3A–3D, and 4A–4D and S7 Fig. (XLSX)

S1 Fig A high level of radiation (100 Gy) destroys spermatogonial compartment of the testes of 3-d-old males. *DSX* [12] pupae were irradiated and observed with confocal microscopy 3 d after adult emergence.
(TIF)

S2 Fig TEP1 is absent from the testes of *7b* and *dsTEP1*-injected males. (A) TEP1 protein levels in the testes of 3- and 14-d-old *7b* and *T4* males that were either not injected or injected with *dsLacZ* or with *dsTEP1* on day 1 after emergence. Testes’ protein extracts were immunoblotted with anti-TEP1 antibodies and anti-α-actin antibody as a protein loading control. (B,C) One-d-old *DSX* males were injected with dsRNA. Two d later, the testes were dissected, stained for TEP1 by immunofluorescence analysis using anti-TEP1 polyclonal antibodies, and observed using confocal microscopy. TEP1 was detected in the testes of *dsLacZ*-injected males (B–B’) but not in *dsTEP1*-injected males (C–C’). (D) Testes from 3-d-old *7b* (TEP1-depleted) males were dissected, stained for TEP1 by immunofluorescence analysis using anti-TEP1 polyclonal antibodies, and observed using confocal microscopy. TEP1 signal was not detected in the testes of *7b* males.
(TIF)

S3 Fig The presence of HPX2 and LRIM1 in the testes. (A) HPX2 (red) and (B) LRIM1 (red) are detected in the cells surrounding the testes. Nuclei are colored by DAPI (blue). *T4* males were dissected, stained with antibody against HPX2 or LRIM1, and observed using confocal microscopy.
(TIF)

S4 Fig DsRNA injection efficiently depletes LRIM1, HPX2, and TEP1 in mosquitoes. One-d-old males were injected with dsRNA, and 2 d later, their hemolymph was extracted for immunoblotting analyses. Injection of *dsLacZ* was used as a negative control. A hemolymph-borne enzyme, prophenoloxidase 2 (PPO2), served as a protein loading control. (A) Silencing of *LRIM1* reduces LRIM1 protein level, while silencing of *LRIM1* and *HPX2* does not affect protein levels of full-length TEP1 (TEP1-F). Note that in the absence of LRIM1, TEP1-cut is no longer detected. (B) Silencing of *HPX2* significantly reduces HPX2 protein levels.
(TIF)

S5 Fig TEP1 is depleted from the hemolymph of control and irradiated (40 Gy) *7b*; *DSX* heterozygous males. Hemolymph of F_1_ males from the reciprocal crosses between *DSX* and *7b* was extracted on the day of emergence for immunoblotting analyses using anti-TEP1 antibodies. A hemolymph-borne enzyme, PPO2, served as a protein loading control.
(TIF)

S6 Fig TEP1 protein levels in the hemolymph are identical among the three *TEP1*-homozygous lines and depleted in the F_1_ progeny of reciprocal crosses between *7b* and the *TEP1*-homozygous lines. Hemolymph was extracted from male mosquitoes on the day of emergence for immunoblotting analyses. A hemolymph-borne enzyme, PPO2, served as a protein loading control.
(TIF)

S7 Fig TEP1 depletion does not affect bacterial loads in male sexual organs. Bacterial loads were gauged in the testes and MAGs of control (*T4*) and TEP1-depleted (*7b*) 1- and 13-d-old males by quantitative PCR of the conserved bacterial *16S* rRNA gene. Larvae of control and TEP1-depleted mosquitoes were raised in the same water and separated at the adult stage according to the expression of fluorescence markers. Three biological repetitions were conducted, each represented by a dot. Fold-change differences in bacterial loads between control and TEP1-depleted mosquitoes are shown. Statistical analysis was performed by two-way ANOVA tests summarized in the tables below the graph. Data used to make this figure can be found in S1 Data.
(TIF)

S8 Fig Comparison of the mosquito complement-like and the mammalian alternative complement activation pathways. Unlike the classical or lectin pathways, in which complement activation is directed to self and nonself surfaces modified by antibody or lectin, respectively, the activation of the alternative pathway is thought to result from a constant spontaneous activation of C3, amplified at the surfaces by the C3b convertase [47]. Conserved requirement for reactive nitrogen species (RNS) in the activation of the mosquito complement-like system revealed here led us to speculate that nitration may play an equally important role in targeting the complement activation to self and nonself in mammals. The mosquito complement-like pathway was designed following the previous studies [5–7,41,48,49].
(TIF)

S1 Table TEP1 genotyping by nested PCR and RFLP. (DOCX)

S2 Table Primers and probes used for qRT-PCR. (DOCX)

## Abbreviations

ANOVA: analysis of variance
APL1C: *Anopheles Plasmodium*-responsive leucine-rich protein 1C
dsRNA: double-stranded RNA
GFP: green fluorescent protein
GSC: germline stem cell
Gy: gray
HPX2: heme peroxidase 2
HPX15: heme peroxidase 15
LRIM1: leucine-rich immune protein 1
MAG: male accessory gland
NOX5: NADPH oxidase 5
PPO2: prophenoloxidase 2
qRT-PCR: quantitative reverse transcription PCR
RFLP: restriction fragment length polymorphism
RNS: reactive nitrogen species
RPL19: ribosomal protein L19
SE: standard error
SEM: standard error of the mean
SSC: somatic stem cell
TEP1: thioester-containing protein 1
TUNEL: TdT-mediated dUTP nick-end labeling
