# Supplementary figures and images for: A New Role of the Mosquito Complement-like Cascade in Male Fertility in Anopheles gambiae
Source: PLoS Biol. 2015 Sep 22;13(9):e1002255. doi: 10.1371/journal.pbio.1002255 (PMC4579081; doi:10.1371/journal.pbio.1002255)

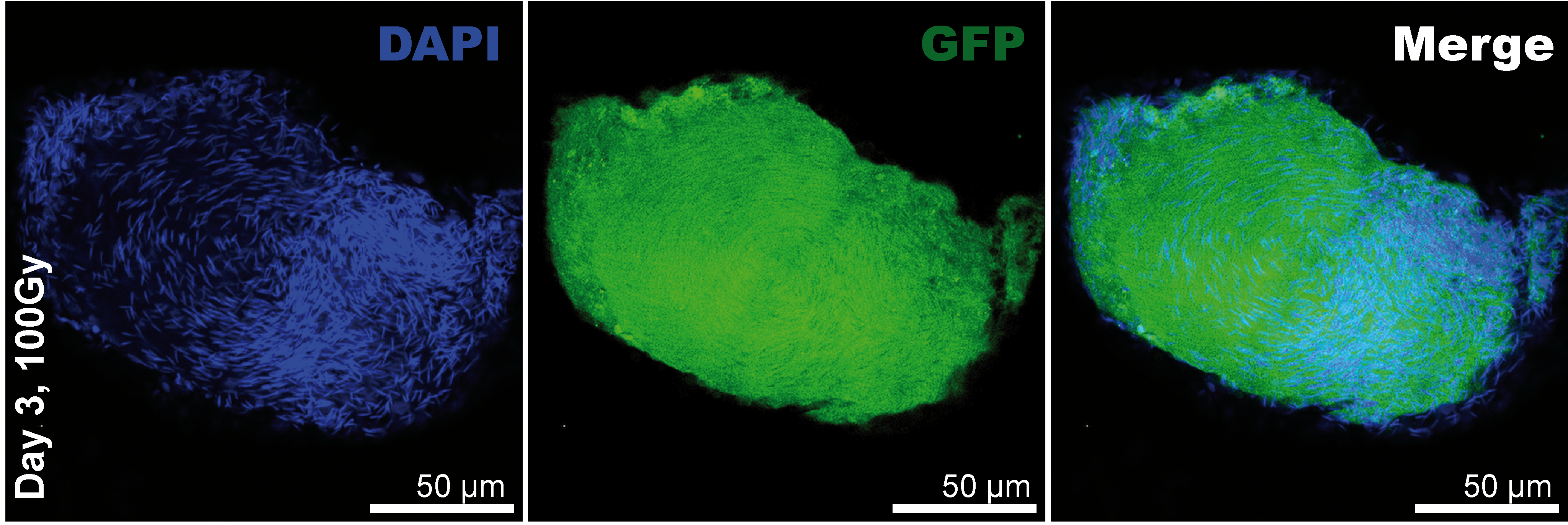

Supplement: S1 Fig — DSX [12] pupae were irradiated and observed with confocal microscopy 3 d after adult emergence. (TIF) [file pbio.1002255.s002.tif]

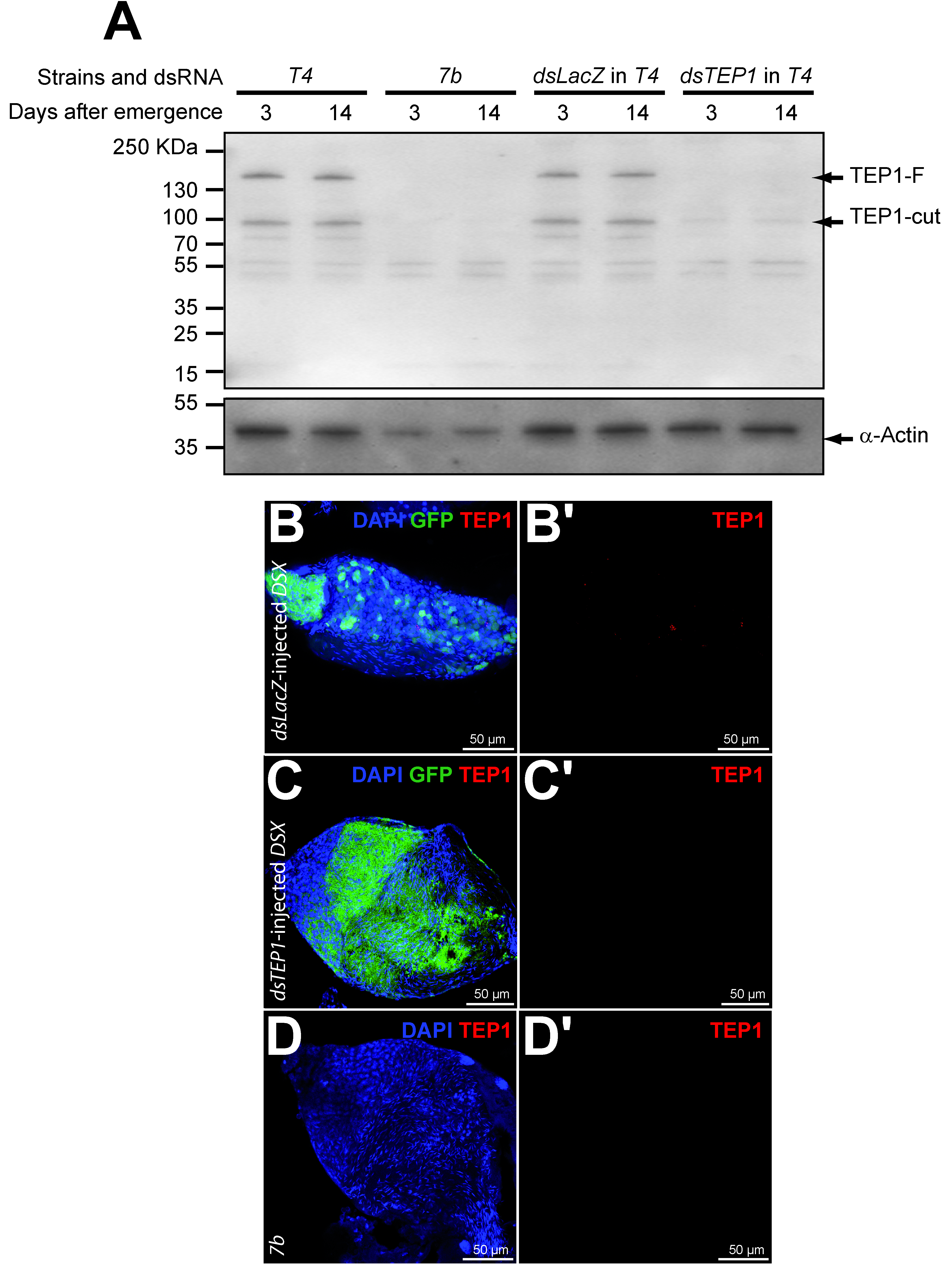

Supplement: S2 Fig — (A) TEP1 protein levels in the testes of 3- and 14-d-old 7b and T4 males that were either not injected or injected with dsLacZ or with dsTEP1 on day 1 after emergence. Testes’ protein extracts were immunoblotted with anti-TEP1 antibodies and anti-α-actin antibody as a protein loading control. (B,C) One-d-old DSX males were injected with dsRNA. Two d later, the testes were dissected, stained for TEP1 by immunofluorescence analysis using anti-TEP1 polyclonal antibodies, and observed using confocal microscopy. TEP1 was detected in the testes of dsLacZ-injected males (B–B’) but not in dsTEP1-injected males (C–C’). (D) Testes from 3-d-old 7b (TEP1-depleted) males were dissected, stained for TEP1 by immunofluorescence analysis using anti-TEP1 polyclonal antibodies, and observed using confocal microscopy. TEP1 signal was not detected in the testes of 7b males. (TIF) [file pbio.1002255.s003.tif]

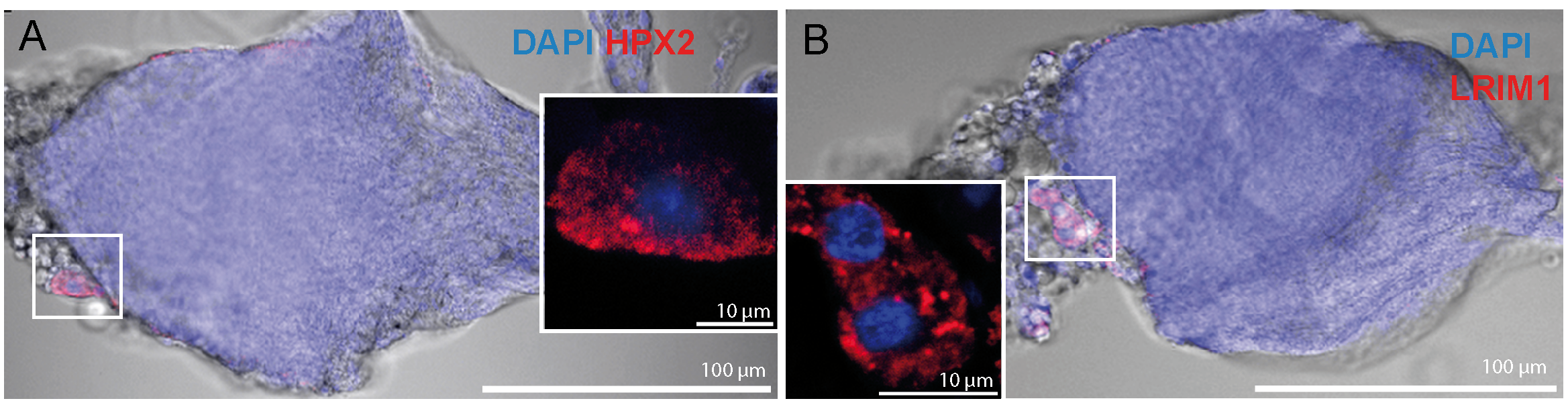

Supplement: S3 Fig — (A) HPX2 (red) and (B) LRIM1 (red) are detected in the cells surrounding the testes. Nuclei are colored by DAPI (blue). T4 males were dissected, stained with antibody against HPX2 or LRIM1, and observed using confocal microscopy. (TIF) [file pbio.1002255.s004.tif]

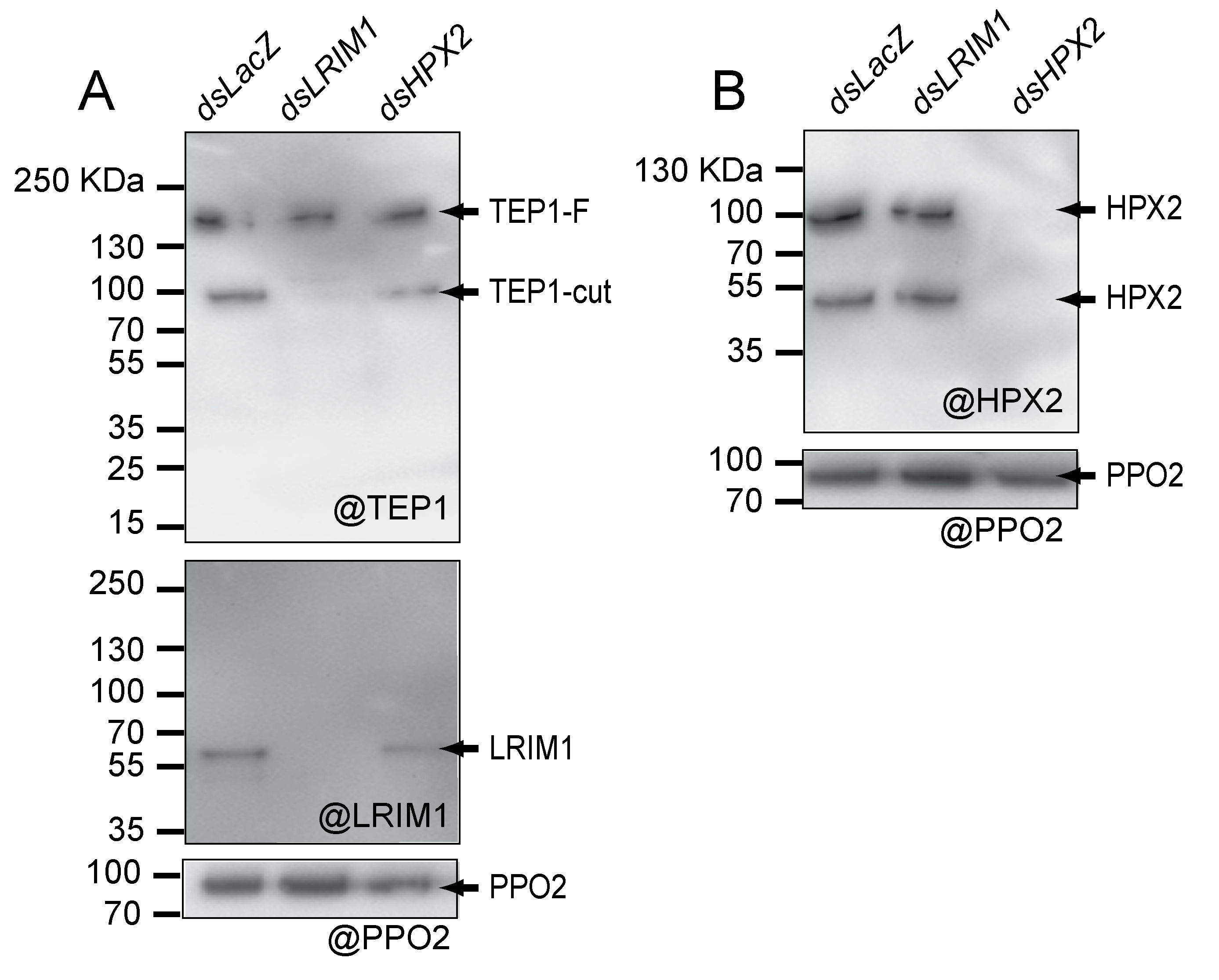

Supplement: S4 Fig — One-d-old males were injected with dsRNA, and 2 d later, their hemolymph was extracted for immunoblotting analyses. Injection of dsLacZ was used as a negative control. A hemolymph-borne enzyme, prophenoloxidase 2 (PPO2), served as a protein loading control. (A) Silencing of LRIM1 reduces LRIM1 protein level, while silencing of LRIM1 and HPX2 does not affect protein levels of full-length TEP1 (TEP1-F). Note that in the absence of LRIM1, TEP1-cut is no longer detected. (B) Silencing of HPX2 significantly reduces HPX2 protein levels. (TIF) [file pbio.1002255.s005.tif]

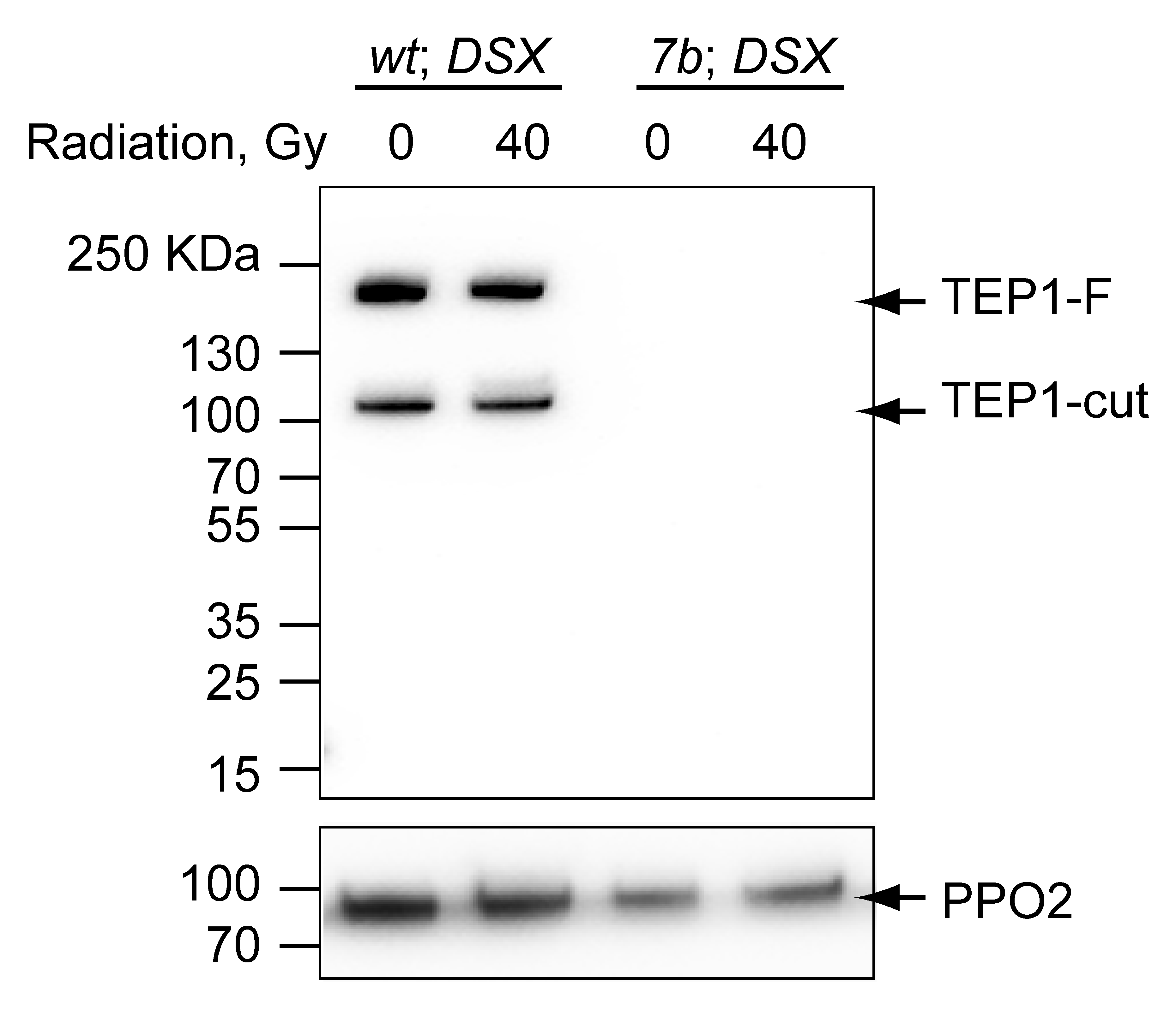

Supplement: S5 Fig — Hemolymph of F1 males from the reciprocal crosses between DSX and 7b was extracted on the day of emergence for immunoblotting analyses using anti-TEP1 antibodies. A hemolymph-borne enzyme, PPO2, served as a protein loading control. (TIF) [file pbio.1002255.s006.tif]

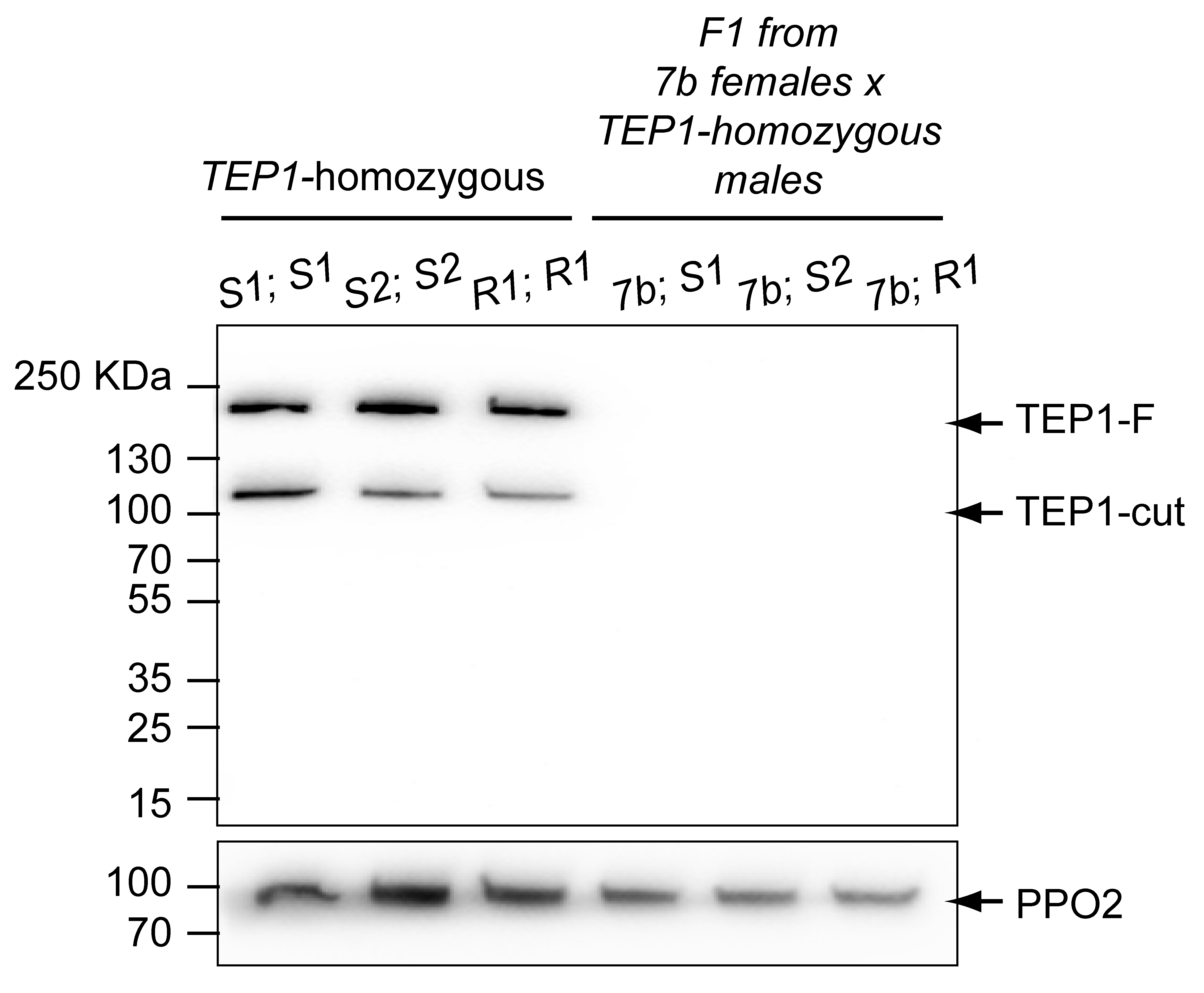

Supplement: S6 Fig — Hemolymph was extracted from male mosquitoes on the day of emergence for immunoblotting analyses. A hemolymph-borne enzyme, PPO2, served as a protein loading control. (TIF) [file pbio.1002255.s007.tif]

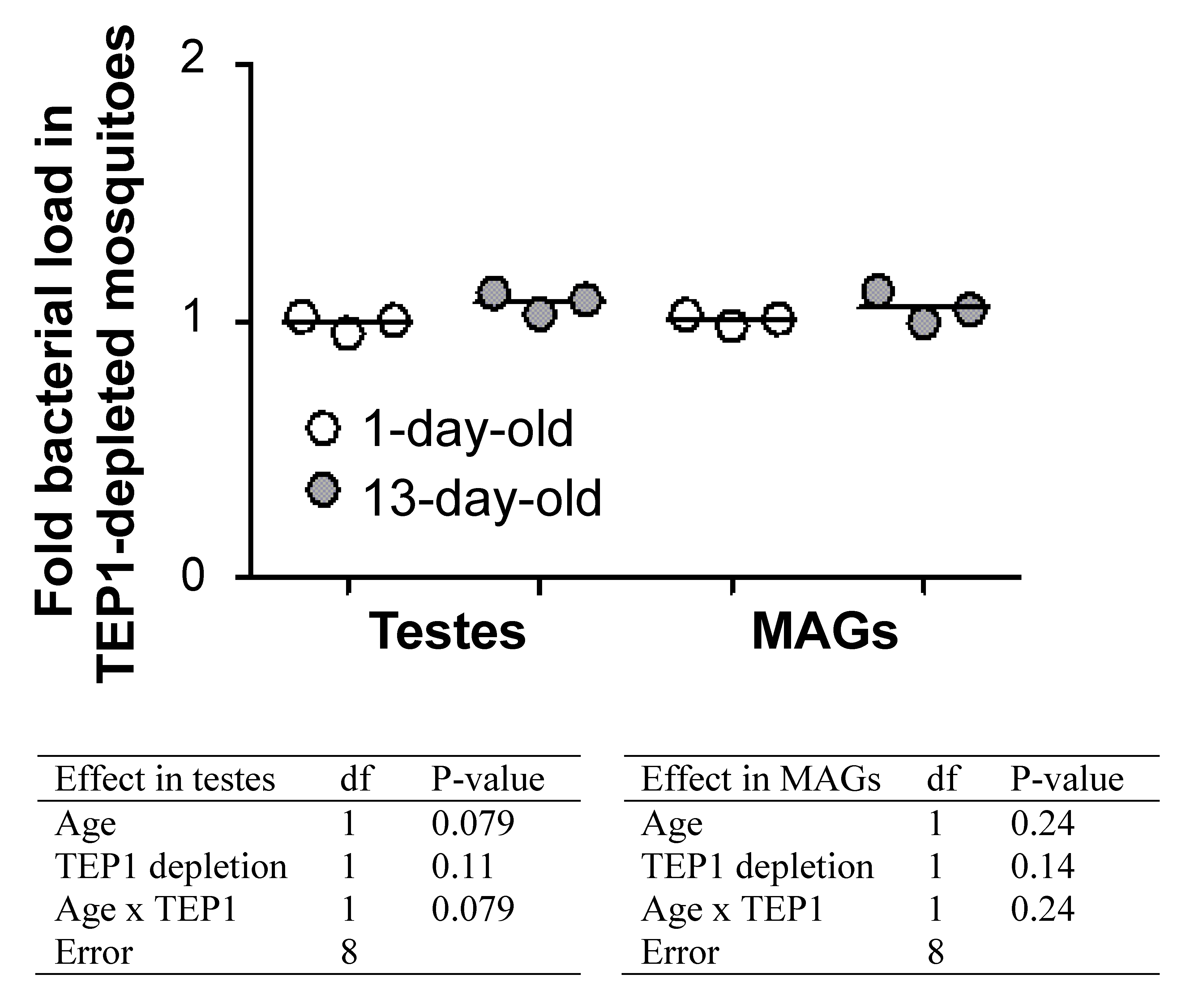

Supplement: S7 Fig — Bacterial loads were gauged in the testes and MAGs of control (T4) and TEP1-depleted (7b) 1- and 13-d-old males by quantitative PCR of the conserved bacterial 16S rRNA gene. Larvae of control and TEP1-depleted mosquitoes were raised in the same water and separated at the adult stage according to the expression of fluorescence markers. Three biological repetitions were conducted, each represented by a dot. Fold-change differences in bacterial loads between control and TEP1-depleted mosquitoes are shown. Statistical analysis was performed by two-way ANOVA tests summarized in the tables below the graph. Data used to make this figure can be found in S1 Data. (TIF) [file pbio.1002255.s008.tif]

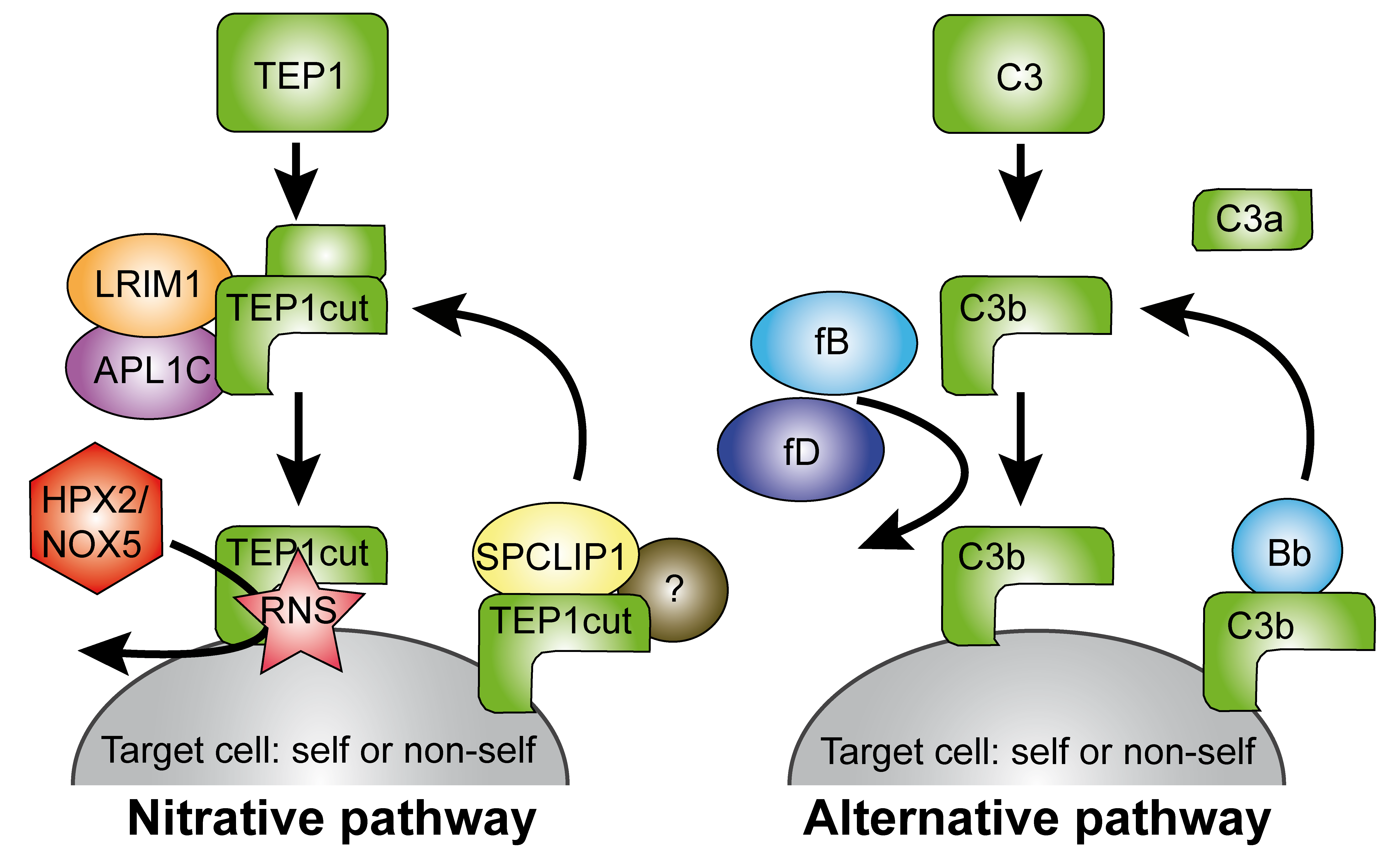

Supplement: S8 Fig — Unlike the classical or lectin pathways, in which complement activation is directed to self and nonself surfaces modified by antibody or lectin, respectively, the activation of the alternative pathway is thought to result from a constant spontaneous activation of C3, amplified at the surfaces by the C3b convertase [47]. Conserved requirement for reactive nitrogen species (RNS) in the activation of the mosquito complement-like system revealed here led us to speculate that nitration may play an equally important role in targeting the complement activation to self and nonself in mammals. The mosquito complement-like pathway was designed following the previous studies [5–7,41,48,49]. (TIF) [file pbio.1002255.s009.tif]
