# Supplementary material for: A New Role of the Mosquito Complement-like Cascade in Male Fertility in Anopheles gambiae
Source: PLoS Biol. 2015 Sep 22;13(9):e1002255. doi: 10.1371/journal.pbio.1002255 (PMC4579081; doi:10.1371/journal.pbio.1002255)
Supplement: S1 Table — (DOCX) [file pbio.1002255.s010.docx]

| **TEP1 genotype** | **Fragment sizes (bp)** | | |
| --- | --- | --- | --- |
|  | ***Bam* HI** | ***Hind* III** | ***Bse* NI** |
| ***R1/R1*** | 399  365 | 764 | 764 |
| ***S1/S1*** | 764 | 657  108 | 537  227 |
| ***S2/S2*** | 764 | 657  108 | 764 |
| ***R1/S1*** | 764  399  365 | 764  657  108 | 764  537  227 |
| ***R1/S2*** | 764  399  365 | 764  657  108 | 764 |
| ***S1/S2*** | 764 | 657  108 | 764  537  227 |
