# Supplementary material for: A New Role of the Mosquito Complement-like Cascade in Male Fertility in Anopheles gambiae
Source: PLoS Biol. 2015 Sep 22;13(9):e1002255. doi: 10.1371/journal.pbio.1002255 (PMC4579081; doi:10.1371/journal.pbio.1002255)
Supplement: S2 Table — (DOCX) [file pbio.1002255.s011.docx]

| **Gene name** | **Gene ID** | **Primers and probes** | **Sequence** | **Reference** |
| --- | --- | --- | --- | --- |
| Universal bacteria *16S rRNA* |  | AG1528 forward  AG1529 reverse | 5’-*TCCTACGGGAGGCAGCAGT*-3’  5’-*GGACTACCAGGGTATCTAATCCTGTT*-3’ | [9] |
| *TEP1*S* | AGAP010815 | AG486 forward  AG487 reverse  TaqMan probe | 5’-*ATACGGATCTCAGCTATACCAAATCG*-3’  5’-*TGCGGGCCTTTATGAGAAAA*-3’  5’-FAM*-TCCGAAGGTTGGTGTTC*-MGB-3’ | [4] |
| *TEP1*R* | AGAP010815 | AG484 forward  AG485 reverse  TaqMan probe | 5’-*ATACGGATCTCAGCTACACCAAATC*-3’  5’-*GCTTGCGGGCCTTGATG*-3’  5’-FAM-*TGAGCGTTCCTCCAAAA-*MGB-3’ | [10] |
| *RPL19* | AGAP004422 | AG490 forward  AG491 reverse  TaqMan probe | 5’-*CCAACTCGCGACAAAACATTC*-3’  5’-*ACCGGCTTCTTGATGATCAGA*-3’  5’-VIC-*CAAACTGATCAAGGATG-*MGB-3’ |  |
